# Right Kocher’s incision: a feasible and effective incision for right hemicolectomy: a retrospective study

**DOI:** 10.1186/1477-7819-10-101

**Published:** 2012-06-07

**Authors:** Theodosios Theodosopoulos, Anneza I Yiallourou, Nicolaos Dafnios, George Polymeneas, Ioannis Papaconstantinou, Chrysoula Staikou, Ioannis Vassiliou, Vassilis Smyrniotis, Alexios Fotopoulos

**Affiliations:** 1 2nd Department of Surgery, Aretaieion Hospital, University of Athens, Athens, Greece; 2 Department of Anesthesiology, Aretaieion Hospital, University of Athens, Athens, Greece; 3 2nd Department of Surgery, Aretaieion Hospital, 76, Vasilissis Sofias Avenue, Athens, 11528, Greece

**Keywords:** Colonic adenocarcinoma, Midline incision, Right Kocher’s incision, Right hemicolectomy

## Abstract

**Background:**

The choice of surgical incision is determined by access to the surgical field, particularly when an oncological resection is required. Special consideration is also given to other factors, such as postoperative pain and its sequelae, fewer complications in the early postoperative period and a lower occurrence of incisional hernias. The purpose of this study is to compare the right Kocher’s and the midline incision, for patients undergoing right hemicolectomy, by focusing on short- and longterm results.

**Methods:**

Between 1995 and 2009, hospital records for 213 patients that had undergone a right hemicolectomy for a right- sided adenocarcinoma were retrospectively studied. 113 patients had been operated via a Kocher’s and 100 via a midline incision. Demographic details, operative data (explorative access to the peritoneal cavity, time of operation), recovery parameters (time with IV analgesic medication, time to first oral fluid intake, time to first solid meal, time to discharge), and oncological parameters (lymph node harvest, TNM stage and resection margins) were analyzed. Postoperative complications were also recorded. The two groups were retrospectively well matched with respect to demographic parameters and oncological status of the tumor.

**Results:**

The median length of the midline incision was slightly longer (12 vs. 10 cm, p < 0.05). The duration of the surgery for the Kocher’s incision group was significantly shorter (median time 70 vs 85 min, p < 0.001). In three patients we performed wedge resection of liver metastasis and in one patient we performed a typical right hepatectomy that lasted 190 min. No major operative complications were noted. There was no immediate or 30- day postoperative mortality. The Kocher’s incision group had a significantly shorter hospital stay (median time 5 vs 8 days). All patients underwent wide tumor excision and clear resection margins were obtained in all cases. No significant difference was noted regarding analgesia requirements and early postoperative complications. Late postoperative complications included 2 incisional hernias and three patients presented with one episode of obstructive ileus, that resolved conservatively.

**Conclusions:**

The Kocher’s incision approach for right- sided colon cancer is technically feasible, safe and overall very well tolerated. It can achieve the same standards of tumor resection and surgical field accessibility as the midline approach, while reducing postoperative recovery.

## Background

The choice of surgical incision is determined by access to the surgical field, particularly when an oncological resection is involved. Special consideration is also given to other factors, such as postoperative pain and its sequelae, fewer complications in the early postoperative period and a lower occurrence of incisional hernias [1-3]. A proper surgical incision should also be extensible, permitting any probable enlargement when required, so that a thorough exploration of the abdominal cavity could be achieved. With the advent of laparoscopic surgery, interest in the influence of less invasive techniques and smaller incisions has increased. Several studies have suggested that transverse incisions could be associated with less postoperative pain, less pulmonary complications, without limitating the access to the abdomen [4-10].

We herein report our results from a retrospective well- matched case control study on a series of patients who underwent right hemicolectomies via a right subcostal laparotomy (Kocher’s incision) or a midline incision for right sided colon cancer.

## Methods

Between January 1995 and December 2009, hospital records for patients who had undergone a right hemicolectomy for a right- sided colonic carcinoma in our department, were retrospectively studied. All of the operations were performed or supervised by a single surgeon. There were a total of 213 patients fulfilling our inclusion criteria. Only elective and curative procedures (defined as no macroscopic residual tumor at the end of the operation) were included. Patients with grossly disseminated carcinoma, tumor fixation to adjacent structures, generalized peritoneal metastasis and recurrent tumor were excluded. Of the 213 patients, 113 patients had undergone a right hemicolectomy via a right subcostal (Kocher’s) incision, identical as the one indicated for an open cholecystectomy and 100 via a midline incision. The two groups were well matched with regards to demographic details (age, sex, body mass index), comorbidities, ASA grading, as well as, oncological status of the tumor (TNM stage, nodes harvested).

Preoperative investigation including full colonoscopy as well as tumor staging with ultrasound or computed tomography was carried out. A right sided colon carcinoma was diagnosed preoperatively in all cases. All patients were given standard bowel preparation on the day prior to surgery and preoperative antibiotic prophylaxis with 2 gr cefotixin and 0.5 gr metronidazole, as well as low molecular weight heparine perioperatively. They were placed on the operating table in the supine position. The right subcostal incision was carried out through the subcutaneous fat to the surface of the external oblique aponeurosis which was divided in the direction of its fibers, and then through the internal oblique and transversus abdominis. Medially the anterior rectal sheath was partially divided. The peritoneal cavity was entered and followed by a typical right hemicolectomy, identically as in operations through the traditional midline incision, without compromising any of the oncological standards of tumor resection. Midline incisions were closed with a single- layer technique and the right subcostal incisions were closed with a two- layer technique using continuous nonabsorbable suture (Nylon- loop).

Postoperatively, patients were given patient- controlled analgesia (PCA) until pain was adequately controlled with simple oral analgesia and oral fluids and diet as tolerated. Wound complications such as hematoma, wound infection with purulent secretion and positive bacteriology were all recorded. General complications, such as atelectacis, urinary tract infections and ileus were defined by radiological and clinical measures. Patients were discharged when they were sufficiently mobile with minimal pain, when they could assume a full diet, and when their bowels were working normally. With regards to late postoperative complications the incidence of incisional hernias in the two groups was recorded.

For each patient we analyzed demographic details, operative data (explorative access to the peritoneal cavity and time of operation), recovery parameters (time with IV analgesic medication, time to first oral fluid intake, time to first solid meal, time to discharge), oncological parameters (lymph node harvest, TNM stage and resection margins), early and late complications.

Results for both incision groups were compared using SPSS Statistical Software (SPSS, Chicago, USA). Statistical analysis was carried out using the Mann- Whitney *U* test. Results were considered significantly different if p < 0.05.

## Results

A total of 213 patients over a period of 14 years fulfilling our criteria were included in our study. There was no statistically significant difference concerning demographic features between the two groups (Table 1). The median length of the midline incision was slightly longer than the right subcostal incision (12 cm vs. 10 cm, p < 0.05). The duration of the surgery ranged from 52 to 100 min (median time 70 min) for the right subcostal incision. Explorative access to the peritoneal cavity through the right subcostal incision was proven excellent, especially on the matter of liver inspection. It was easy to divide the lateral peritoneal attachments and divide the gastrocolic ligament. After adequate mobilization of the right colon, it was carefully brought through the laparotomy wound, with its mesentery. Next, the resection of the affected right colon with its whole lymph node-bearing mesentery and the corresponding portion of the greater omentum was performed and followed by an end-to-end ileocolonic anastomosis. Three patients underwent segmentectomies of the right hepatic lobe, prior to the right hemicolectomy for a preoperatively diagnosed metastatic lesion, whereas one patient underwent a typical right hepatectomy. No major operative complications were noted. Blood transfusion was required in a female patient, aged 91 years. There was no immediate or 30- day postoperative mortality. On the other hand, the duration of surgery in the midline group was significantly longer (duration of operation 67 to 140 min, median time 85 min, p < 0.001). There was also no statistically significant difference concerning oncological outcomes between the two groups (Table 1). All patients underwent wide tumor excision. According to the histopathological reports, clear resection margins were obtained in all cases (minimum length of resection margins was 5.7 cm distally for the right subcostal incision group and 5.8 cm for the midline incision group), whereas the median number of lymph nodes harvested was 14 for both groups. TNM stage between the two groups showed no statistically significant difference.

**Table 1:** Demographic and tumour resection data for each of the incision groups

	**Midline incision**	**Right Kocher incision**	**p value**
	**(n=100)**	**(n=113)**	
Age, years	65 (53-86)	65 (39-91)	NS
Females, n (%)	53 (53)	60 (53%)	NS
Body mass index (BMI-kg/m^2^)	21 (18–26)	21(17–27)	NS
Incision length, cm	12(10–30)	10 (8–12)	<0.05
Duration of operation, min	85(67–140)	70 (52–100)	<0.001
Nodes harvested, n	14 (5–20)	14 (5–25)	NS
TNM Stage (n and %)			NS
I	18 (18)	21 (18.6)	
II	52 (52)	56(49.5)	
III	28 (28)	32 (28.3)	
IV	2 (2)	4 (3.6)	
Positive resection margins	0	0	NS

^1^Values are median (range), unless otherwise indicated.

^2^NS, not significant.

The postoperative recovery parameters for both groups are as shown in Table 2. There was no significant difference between the two groups with regards to postoperative analgesia requirements. Cessation of PCA was accomplished on the 2^nd^ postoperative day for the right subcostal incision group (range 1 to 5) and on the 3^rd^ postoperative day for the midline incision group (range 2 to 7). None of the postoperative recovery parameters were significantly different between the two groups, with the exception of the median time to discharge. The Kocher incision group had a significantly shorter hospital stay (median time 5 days, range from 2 to 8 days) than the midline incision group (median time 8 days, range from 5 to 16 days).

**Table 2:** Postoperative recovery parameters for patients, by surgical group

	**Midline incision**	**Right Kocher incision**
Oral fluids intake	1 (1–9)	2 (1–9)
Solid diet	3 (2–10)	4 (2–10)
Discontinue PCA	3 (2–7)	2 (1–5)
Bowel movement	3 (3–6)	3 (2–8)
Discharge	8 (5–16)	5 (2–8)

^1^Values are median numbers of days (range) after operation.

^2^There was no significant difference between the two groups, with the exception of median time to discharge.

^3^PCA, patient-controlled analgesia.

There was no 30- day postoperative mortality in either group. Twenty-eight early postoperative complications occurred: chest infection in 13 patients (7 with midline incision, 6 with the Kocher incision), urinary tract infection in 9 patients (5 from the Kocher’s incision group), wound infection in 4 patients (2 from the midline incision group) that did not prolong hospital stay time, since their treatment was completed in an outpatient basis and prolonged ileus in two patients (one from each group) that did not require surgical intervention. There was no significant difference in terms of early postoperative complications between the two groups.

The follow- up period for both of the groups ranged from 2 to 15 years. With regards to late postoperative complications, two patients from the Kocher incision group presented with incisional hernias (1.8%), whereas six patients from the midline incision group (8%) had this complication.

## Discussion

Despite several experimental and clinical studies, there is still no consensus on which is the ideal incision for elective major abdominal surgeries. Transverse and midline incisions are both commonly used for laparotomy. Both incisions have specific effects on abdominal wall function, predisposing to postoperative differences in clinical parameters such as pulmonary function, pain, wound healing and the risk of incisional hernia. With regards to fewer early postoperative complications, transverse incisions especially for right colon surgery are advocated to be superior to midline incisions by several authors [1-3,11].

With the introduction of laparoscopic colorectal surgery and its advantages (less postoperative pain, shorter hospitalization, rapid return of bowel function and better cosmesis) the need for improvements in conventional open surgery has emerged. Over the last decade, a number of studies have introduced minilaparotomy as a feasible method for approaching colon cancer. The advantages of mini- incisions include lower cost, faster completion of procedure, reduced bulkiness of equipment and the possibility of exploring the entire peritoneal cavity without loss of tactile sensation [12-14]. It is quite interesting, though, that in retrospective studies which compared the effects of laparoscopic- assisted colectomies and open colectomies through right transverse skin incisions for right- sided colon cancer specifically, no statistically significant differences were noted with regards to short- term surgical outcomes and oncological parameters [15].

In our series, we compared two standard incisions for the same procedure of right hemicolectomy for colonic carcinoma. We accessed the data of 213 patients, 100 with midline incision and 113 with a right subcostal incision.

Based on numerous studies demonstrating the positive influence of a right subcostal (Kocher) laparotomy on the postoperative recovery of the surgical patient [3,10,12,13,16,17], we performed 113 right hemicolectomies. Exposure of the surgical field and accessibility of the abdominal cavity were proven excellent. Technically, two major advantages were noted in comparison to the midline or the other transverse incisions. First, the convenience for accurate, proper full liver mobilisation with the ability of performing an intraoperative US scan and the extensiblility, if needed, up to a bilateral subcostal incision for conducting a liver resection in the presence of positive intraoperative findings. In our series, three patients underwent segmentectomies of the right hepatic lobe, prior to the right hemicolectomy for a preoperatively diagnosed metastatic lesion. The complete operations were carried out through extended right subcostal incisions, which were proven safe and convenient for both procedures. Secondly, we experienced easy mobilization of the hepatic flexure and facile conduction of the ileocolonic anastomosis, since after colonic resection the stamp of the transverse colon lies almost always exactly under the surgical trauma. The right subcostal incision length was significantly smaller, but this difference was minimal. In a retrospective study reported by Donatti et al. [2], a 10- cm difference in midline incision length was recorded. We also noted that the right subcostal incision group had shorter operating times comparing with the midline incision group.

Interestingly, the analgesic requirements, measured as the length of time required for discontinuation of patient- controlled analgesia, were similar between the two groups, but slightly better for the right subcostal incision group. Pain assessment is quite difficult to quantify and is dependent on local ward practices than the amount of pain actually experienced [7].

Impairment of postoperative pulmonary function has been documented in several studies [4,5,9,10], showing slower recovery and more incisional pain perception after a midline incision.

Our results showed that all postoperative recovery parameters were similar for both groups, with one significant exception. The Kocher incision group had a shorter hospital stay than the midline incision group. No difference was found with regards to early postoperative complications. On the other hand, there was a significant difference in terms of incisional hernias between the two groups (1.8% for the right subcostal group vs. 8% for the midline incision group). The incidence of incisional hernias is thought to be influenced by immediate postoperative changes. In a study published by Pollock et al. [18], 94% patients who presented with an incisional hernia 3 years after surgery had been found to have radiologic evidence of dehiscence of intraoperative fixed metallic clips as early as 1 month postoperatively.

Nor obesity or advanced TNM stage were proven contraindications in order to successfully perform the operation through a right subcostal incision. The oncological parameters available were similar. All patients underwent high tie and wide tumor excisions. Clear resection margins were obtained in all cases and the lymph node harvests were no different.

In conclusion, we found that the right subcostal incision approach for right sided colon cancer is technically feasible, safe and overall very well tolerated. The oncological standards of the surgical procedure are not compromised. We were able to demonstrate some important advantages in terms of postoperative recovery and cosmetic results, such as incisional hernias, but more prospective randomized trials must be carried out in order to confirm these advantages. The type of incision for right hemicolectomy still remains a decision based on the surgeon’s preference.

## Conclusions

The short- term outcomes of our study allowed us to conclude that this approach appears to be most satisfying in terms of accessibility, safety and oncological efficacy. Furthermore, it demonstrates important advantages compared to other incisions. The major advantage of the subcostal incision over the upper midline incision is greater lateral exposure and less pain. Upper midline incisions are very painful and restrict pulmonary function, particularly vital capacity, by about 50%. The disadvantage of the subcostal incision is that the operation takes longer, because there are more layers to close. Generally, the subcostal incision heals well. To our knowledge, this is the first report of such series, since other transverse incisions have been utilized for a right hemicolectomy, but not a typical right Kocher incision.

## Competing interests

All of the authors declare that they have no competing interests.

## Authors’ contributions

TT made substantial contribution to analysis and interpretation of data, revised the manuscript critically for important intellectual content and approved the final version. AIY made substantial contribution to acquisition and analysis of data, drafted the manuscript and approved the final version to be published. ND made substantial contribution to conception and design of the study, revised the manuscript critically for important intellectual content and approved the final version. GP made substantial contribution in acquisition and interpretation of data, drafted the manuscript and approved the final version to be published. IP made substantial contribution to analysis and interpretation of data, drafted the manuscript and approved the final version. CS made substantial contribution to analysis and interpretation of data, revised the manuscript critically for important intellectual content and approved the final version. IV participated in the design of the study and its coordination and helped to draft the manuscript. AF made substantial contribution to concept and design of the study, revised the manuscript critically for important intellectual content and approved the final version to be published. All authors read and approved the final manuscript.
